# Genome-wide identification, expression profiling, and protein interaction analysis of the *CCoAOMT* gene family in the tea plant (*Camellia sinensis*)

**DOI:** 10.1186/s12864-024-09972-y

**Published:** 2024-03-04

**Authors:** Yiqing Wang, Tao Wang, Siyu Qi, Jiamin Zhao, Jiumei Kong, Zhihui Xue, Weijiang Sun, Wen Zeng

**Affiliations:** 1 College of Horticulture, Fujian Agriculture and Forestry University, 350002 Fuzhou, China; 2 Anxi College of Tea Science, Fujian Agriculture and Forestry University, 350028 Quanzhou, China

**Keywords:** CCoAOMT, *Camellia sinensis*, Bioinformatics, Gene expression, Interaction networks

## Abstract

**Background:**

The caffeoyl-CoA-*O* methyltransferase (CCoAOMT) family plays a crucial role in the oxidative methylation of phenolic substances and is involved in various plant processes, including growth, development, and stress response. However, there is a limited understanding of the interactions among CCoAOMT protein members in tea plants.

**Results:**

In this study, we identified 10 members of the *CsCCoAOMT* family in the genome of *Camellia sinensis* (cultivar ‘HuangDan’), characterized by conserved gene structures and motifs. These *CsCCoAOMT* members were located on six different chromosomes (1, 2, 3, 4, 6, and 14). Based on phylogenetic analysis, CsCCoAOMT can be divided into two groups: I and II. Notably, the CsCCoAOMT members of group Ia are likely to be candidate genes involved in lignin biosynthesis. Moreover, through the yeast two-hybrid (Y2H) assay, we established protein interaction networks for the CsCCoAOMT family, revealing 9 pairs of members with interaction relationships.

**Conclusions:**

We identified the *CCoAOMT* gene family in *Camellia sinensis* and conducted a comprehensive analysis of their classifications, phylogenetic and synteny relationships, gene structures, protein interactions, tissue-specific expression patterns, and responses to various stresses. Our findings shed light on the evolution and composition of *CsCCoAOMT*. Notably, the observed interaction among CCoAOMT proteins suggests the potential formation of the *O*-methyltransferase (OMT) complex during the methylation modification process, expanding our understanding of the functional roles of this gene family in diverse biological processes.

**Supplementary Information:**

The online version contains supplementary material available at 10.1186/s12864-024-09972-y.

## Background

Tea, the second most consumed beverage globally after water, is beloved by consumers worldwide for its numerous health benefits and distinctive flavor. Tea leaves contain a diverse array of secondary metabolites, including tea polyphenols, purine alkaloids [1], and aromatic compounds [2]. One of these aromatic compounds is lignin, which primarily accumulates in secondary thickening cells [3]. Lignin provides mechanical support to plant cells and tissues, aiding in the transportation of water and nutrients. Additionally, lignin plays a role in various responses to biotic or abiotic stress [4]. However, the accumulation of lignin in tea leaves is not always advantageous, as it negatively affects tenderness. Tenderness directly impacts the grade of tea products and necessitates different processing techniques based on varying levels of tenderness [5]. Therefore, the degree of lignification in tea leaves serves as a crucial reference for grading products and establishing processing parameters [5]. Methylation is a widespread and significant chemical modification in organisms that can alter the biological activity of compounds. (-)-Epigallocatechin gallate (EGCG), the primary type of catechin in tea plants, contributes to the bitter taste of tea and possesses strong antioxidant properties that confer several health benefits [6, 7]. Another methylated derivative of EGCG, (-)-Epigallocatechin 3-*O*-(3-*O*-methyl) gallate (EGCG3″Me), has been found to exhibit stronger anti-allergic and anti-obesity effects than EGCG [8]. Interestingly, recent research has discovered that the genes responsible for lignin and EGCG3"Me biosynthesis in tea plants (*Camellia sinensis*) belong to the caffeoyl-CoA-*O* methyltransferase (CCoAOMT) family [9].

Lignin, an important product of the phenylalanine metabolism pathway, has been extensively studied in numerous plants due to its biosynthesis pathway. It consists of three types of monomers: p-hydroxyphenyl lignin (H lignin), guaiacyl lignin (G lignin), and syringyl lignin (S lignin) [10]. CCoAOMT, classified as class I *O*-methyltransferase (OMT), is a key enzyme involved in lignin biosynthesis, responsible for catalyzing the conversion of Caffeoyl-CoA to Feruloyl-CoA in plants [11]. It transfers the methyl (-CH_3_) group from *S*-adenosyl-L-methionine to the hydroxyl (-OH) group of caffeoyl CoA [12]. CCoAOMT plays a crucial role in G lignin synthesis and provides substrates for S lignin synthesis [13]. Research has indicated that high nitrogen fertilization could lead to reduced lignin deposition and content [14]. Additionally, the content and composition of lignin are influenced by the levels of key enzymes, such as CCoAOMT, in the biosynthetic pathway. Expression of *CCoAOMT* in *Populus tomentosa* is also affected by external nitrogen content, with different forms and concentrations of nitrogen exerting varying effects on the expression patterns of *PtCCoAOMT* members [15]. Genome analysis results indicate the presence of 11, 9, and 6 *CCoAOMT* genes in *Arabidopsis* [12], *rice* [16], and *poplar* [17], respectively. CCoAOMT consists of 8 conserved motifs labeled as A to H. Motifs A (LVKVGGLIG), B (VAPPDAPLRKY), and C (ALAVDPRIEICM) are universal characteristic sequences found in all OMTs, while motifs D (TSVYPREPEPMKELRELT), E (KLINAKNTMEI), F (PVIQKAGVAHKIEF), G (DFIFVDADKDNY), and H (GDGITLCRR) are specific to CCoAOMT [18].

In 1988, CCoAOMT was found in tissue-cultured cells of carrot and parsley, where it was found to be associated with the defense response of tissue-cultured cells against fungal infection [19]; *CCoAOMT1* in *Arabidopsis* is involved in drought stress resistance by regulating the accumulation of H_2_O_2_ as well as ABA and ROD signaling [20]. Knockout of the gene encoding the CCoAOMT enzyme in *Arabidopsis* resulted in reduced G lignin content and increased S lignin and H lignin [11, 21]. This knockout also led to a hypersensitivity phenotype to salt stress by inhibiting the main root elongation [22]. *ZmCCoAOMT2* in maize can regulate H lignin content and programmed cell death (PCD), playing a significant role in resistance against diseases like necrotic leaf blight (NLB), southern leaf blight (SLB), grey leaf spot (GLS), and other diseases [23]. Recent studies have revealed that CCoAOMT is not only involved in lignin biosynthesis but also the metabolism of erucic acid [11] and the biosynthesis of isorhamnetin [24] by catalyzing the methylation of hydroxycinnamic acid or the flavonoid precursor. In addition, CCoAOMT participates in the biosynthesis of anthocyanins, impacting plant color. For instance, under the catalysis of CCoAOMT, cyanidin produces paeoniflorin, resulting in the purple-red color of immature ‘Tailihong’ *jujube* fruit [25]; CCoAOMT also controls the methylation process of anthocyanins in the grape peel, thereby giving the berry peel a purple color [26, 27]. In tea plants, EGCG3"Me, which is formed after EGCG methylation, exhibits improved water solubility and higher bioavailability compared to EGCG [28], leading to enhanced health benefits. Notably, CsCCoAOMT has been found to possess a novel catalytic function in tea plants, enabling the methylation of EGCG to produce EGCG3"Me [9]. Tea plants are known to be fluoride (F) accumulators, and studies suggest that fluorides affect the accumulation of catechins and lignin in tea plants while inhibiting the activity of phenylalanine ammonia-lyase [29]. As CCoAOMT is an essential regulator in the synthesis pathways of catechins and lignin, its role in multiple stress responses and the production of secondary metabolites in plants has been confirmed. This underscores its significant importance in overall plant growth and development.

However, there is still a lack of research on the evolutionary relationship and functional verification of the *CCoAOMT* family in *Camellia sinensis*. This paper addresses this gap by employing bioinformatics analysis to identify the *CCoAOMT* gene family in the ‘Huang Dan’ genome. Furthermore, we elucidated the evolutionary relationship of CCoAOMT members from different plants, investigated the expression pattern of *CsCCoAOMT* in response to fluoride and nitrogen treatments, and detected potential interactions among CsCCoAOMT members using yeast two-hybrid assays. These results significantly contribute to our understanding of the *CCoAOMT* genes and provide a new perspective for future investigation into the characterization of these genes.

## Results

### Genome-wide identification of the *CsCCoAOMT* gene family

The *CsCCoAOMT* gene family was identified in the *Camellia sinensis* (cultivar ‘HuangDan’) genome through HMM searches. Initially, 13 putative *CsCCoAOMT* genes were obtained, and candidate genes were further analyzed using the CDD and SMART databases to remove incomplete sequences. Eventually, 10 *CCoAOMT* genes were identified and named *CsCCoAOMT1*–*CsCCoAOMT10*.

Bioinformatics analysis of *CsCCoAOMT* showed variations in ORF lengths, protein lengths, molecular weights (MW), and theoretical pI (isoelectric point) values across the identified genes (Table S1). ORF lengths ranged from 702 to 864 bp, protein lengths ranged from 233 aa (CsCCoAOMT6) to 287 aa (CsCCoAOMT4), MWs ranged from 26.29 to 32.13 kDa, and the theoretical pI values ranged from 5.28 to 8.96. Except for CsCCoAOMT4, all proteins were predicted to be stable proteins, and no signal peptides or TMHs (transmembrane helices) were predicted in any of the proteins. Subcellular localization prediction indicated that CsCCoAOMT4 and the remaining CsCCoAOMT8 were located in chloroplasts.

### Chromosome location and homology analysis of the *CsCCoAOMT* gene family

**Fig. 1:**
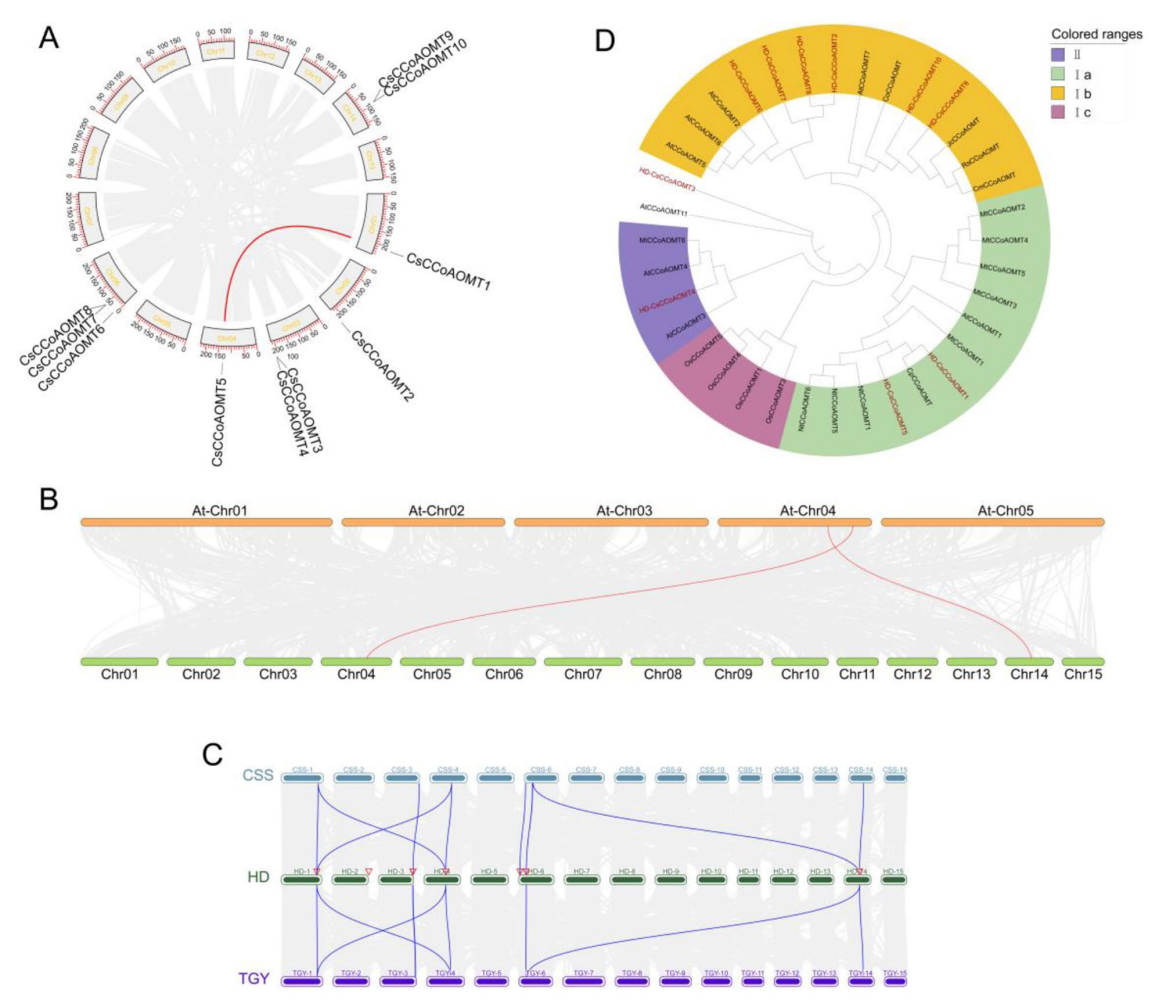
Chromosomal distribution, synteny analysis, and Phylogenetic analysis of CsCCoAOMT. (**A**) The chromosomal distribution and collinearity analysis of *CsCCoAOMT* in the ‘Huangdan’ genome. (**B**) The interspecies synteny analysis of *CsCCoAOMT* in ‘HuangDan’ associated with *Arabidopsis*. (**C**) Synteny analysis of CsCCoAOMT in ‘HuangDan’, ‘ShuChaZao’and ‘TieGuanYin’ cultivars. (**D**) Phylogenetic analysis of CsCCoAOMT and CCoAOMT from other plants

The chromosomal location of genes is determined by previous evolutionary events. Thus, our investigation revealed that *CsCCoAOMT* members were randomly distributed across six chromosomes. Among them, chromosome 6 contained three genes, chromosomes 3 and 14 had two genes each, and chromosomes 1, 2, and 4 each had one gene (Fig. 1A).

To elucidate the evolutionary relationships among *CsCCoAOMT* genes, we analyzed the synchrony within the *CsCCoAOMT* family. Our analysis identified only one homologous pair (*CsCCoAOMT1*/*CsCCoAOMT5*). In addition, calculations of the Ka/Ks ratios of *CsCCoAOMT* indicated a value of 0.067 for *CsCCoAOMT1*/*CsCCoAOMT5*, indicating that they have undergone purifying selection.

To gain further insight into the evolutionary relationships of *CsCCoAOMT*, we constructed interspecies comparative syntenic maps involving *Camellia sinensis* (cultivar ‘HuangDan’), *Arabidopsis thaliana*, and two other *Camellia sinensis* cultivars (‘TieGuanYin’ and ‘ShuChaZao’). Analysis of collinearity revealed that 2 *CsCCoAOMT* genes exhibited syntenic relationships with *AtCCoAOMT* (*CsCCoAOMT5*/*AtCCoAOMT1*, *CsCCoAOMT10*/*AtCCoAOMT7*) (Fig. 1B). We found 10 pairs of collinear genes between ‘HuangDan’ and ‘ShuChaZao’, and 9 pairs of collinear genes between ‘HuangDan’ and ‘Tieguanyin’, with slightly higher homology observed between ‘HuangDan’ and ‘ShuChaZao’ (Fig. 1C).

### Phylogenetic analysis of CsCCoAOMT proteins

It identified 8 members of CCoAOMT in *Arabidopsis thaliana*, specifically AtCCoAOMT1-7 and AtCCoAOMT11. Sequence analysis showed significant differentiation between AtCCoAOMT1-7 and AtCCoAOMT11. Therefore, only AtCCoAOMT11 was chosen as the outer group for our analysis [30].

A total of 36 CCoAOMT proteins were used to construct a phylogenetic tree. The results of the phylogenetic analysis demonstrated that all CCoAOMT proteins were divided into two subfamilies (Fig. 1D). In the evolutionary tree, the CCoAOMT proteins were categorized into 2 subclasses: I and II. Group I comprised 3 subbranches: Ia, Ib, and Ic. The Ia branch included CsCCoAOMT1, CsCCoAOMT5, and AtCCoAOMT1, which are typical dicotyledons and have been proven to be involved in lignin biosynthesis. Subbranch Ib consisted of CsCCoAOMT2, CsCCoAOMT6, CsCCoAOMT7, CsCCoAOMT8, CsCCoAOMT9, CsCCoAOMT10, AtCCoAOMT2, AtCCoAOMT5, AtCCoAOMT6, and AtCCoAOMT7. Group II comprised CsCCoAOMT4, AtCCoAOMT3, and AtCCoAOMT4. Group I showed a distant evolutionary relationship with Group II. CsCCoAOMT3, along with AtCCoAOMT11, was classified as an outer group, indicating that CsCCoAOMT3 has a distant evolutionary relationship with other CCoAOMT members. Overall, the cultivar “HuangDan” had a close evolutionary relationship with *Arabidopsis thaliana*. We speculate that the CCoAOMT proteins in *Camellia sinensis* and *Arabidopsis thaliana* do not exhibit obvious differentiation and may share some functional similarities.

### Gene structure and motif composition of the *CsCCoAOMT* gene family

**Fig. 2:**
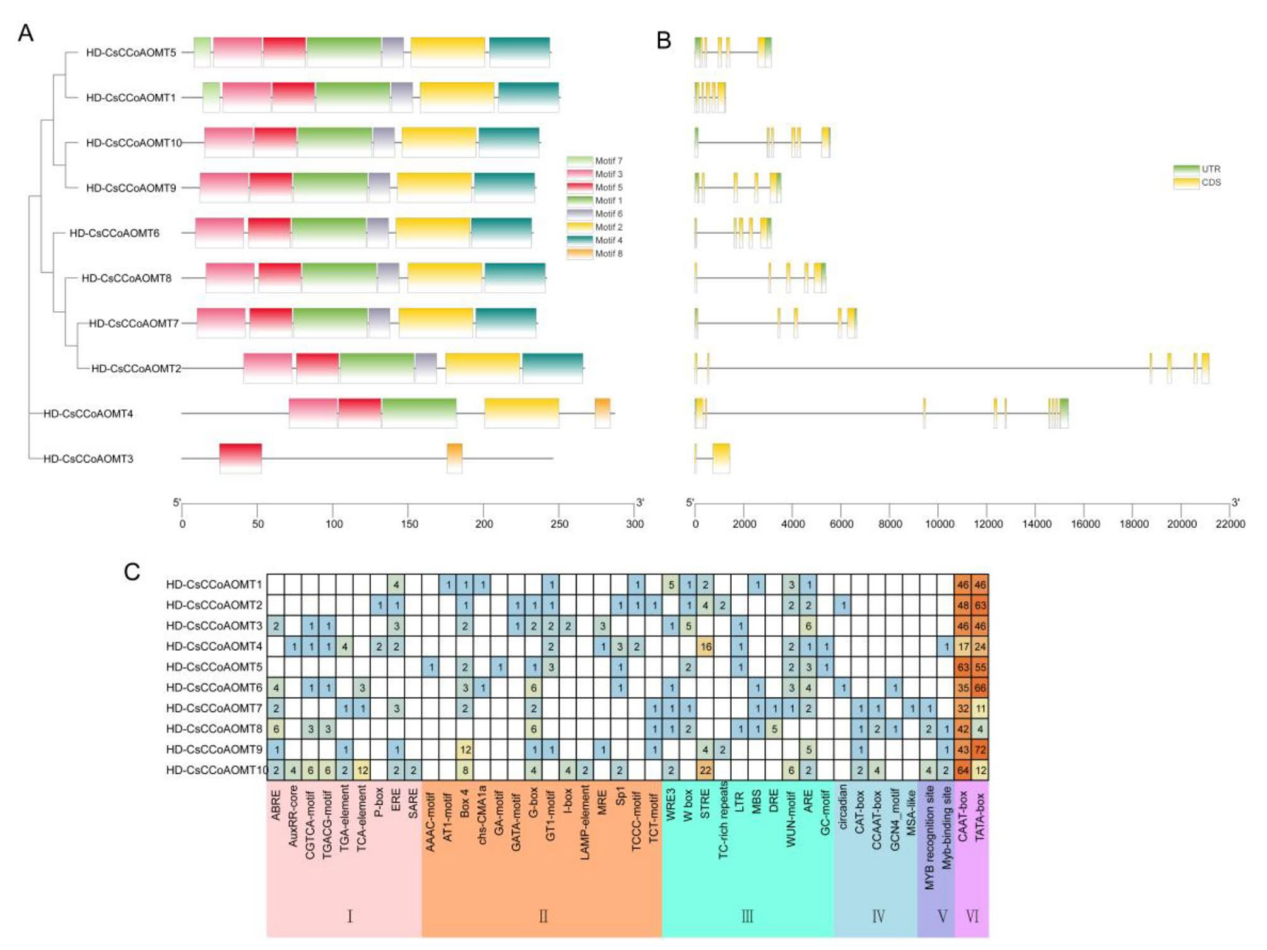
Motifs, exon-intron structures, and cis-acting elements in promoters of *CsCCoAOMT* genes (**A**) Conserved motifs of CsCCoAOMTs. (**B**) The exon-intron structures of *CsCCoAOMT*. (**C**) *cis*-acting elements in promoters of *CsCCoAOMT*. Note I: Phytohormone responsive;II: Light responsive; III: Abiotic stress responsive; IV: Plant growth; V: TF reconition and binding site; VI: Core

To gain a better understanding of the structural characteristics of CsCCoAOMT proteins, the compositions of conserved motifs were analyzed using MEME. The analysis predicted and assigned names to eight conserved motifs, labeled as motifs 1–8. As illustrated in Fig. 2A, all CCoAOMT members in the Ia branch possess motifs 1–7, while those in the Ib branch include motifs 1–6, Group II member CsCCoAOMT4 contains motifs 1, 2, 5, and 8, whereas CsCCoAOMT3 only contains motifs 5 and 8 (Fig. 2A).

We observed variations in the exon-intron distribution patterns among the *CsCCoAOMT* gene family in terms of intron length and exon number. Specifically, *CsCCoAOMT1*, *CsCCoAOMT5*, *CsCCoAOMT6*, *CsCCoAOMT7*, *CsCCoAOMT8*, *CsCCoAOMT9*, and *CsCCoAOMT10* have 5 exons and 4 introns, *CsCCoAOMT6* contains 6 exons and 5 introns, and *CsCCoAOMT4* has 9 exons and 8 introns. Interestingly, *CsCCoAOMT3* exhibits a distinct pattern with only 2 exons, positioning it far from other members in terms of evolutionary relationships (Fig. 2B).

### *Cis*-regulatory elements in the promoters of the *CsCCoAOMT* gene family

We examined the promoter regions by extracting 2000 bp upstream sequences of the 10 *CsCCoAOMT* genes to analyze the *cis*-regulatory elements (Fig. 2C). A total of 33 types of core initiator elements were identified, which we categorized into six groups based on their functions: light response elements, plant hormone response elements, stress response elements, and plant growth and development elements. A large number of light response *cis*-acting elements were found upstream of 10 *CsCCoAOMT* genes, suggesting the regulation of *CsCCoAOMT* by light signals. Moreover, various hormone response elements were discovered, including methyl jasmonate responsiveness element (MeJA), salicylic acid responsiveness element (SA), abscisic acid responsiveness element (ABRE), and auxin responsiveness element (AUX). *CsCCoAOMT3* had 6 ABREs upstream, while *CsCCoAOMT5* had 7 SA elements (Fig. 2C). These results indicate that *CsCCoAOMT* is regulated by multiple hormones. Notably, the upstream regions of the genes *CsCCoAOMT4*, *CsCCoAOMT7*, *CsCCoAOMT8*, *CsCCoAOMT9*, and *CsCCoAOMT10* all contain *MYB* binding sites involved in the regulation of flavonoid biosynthesis.

### Tissue-specific expression patterns of *CsCCoAOMT*

**Fig. 3:**
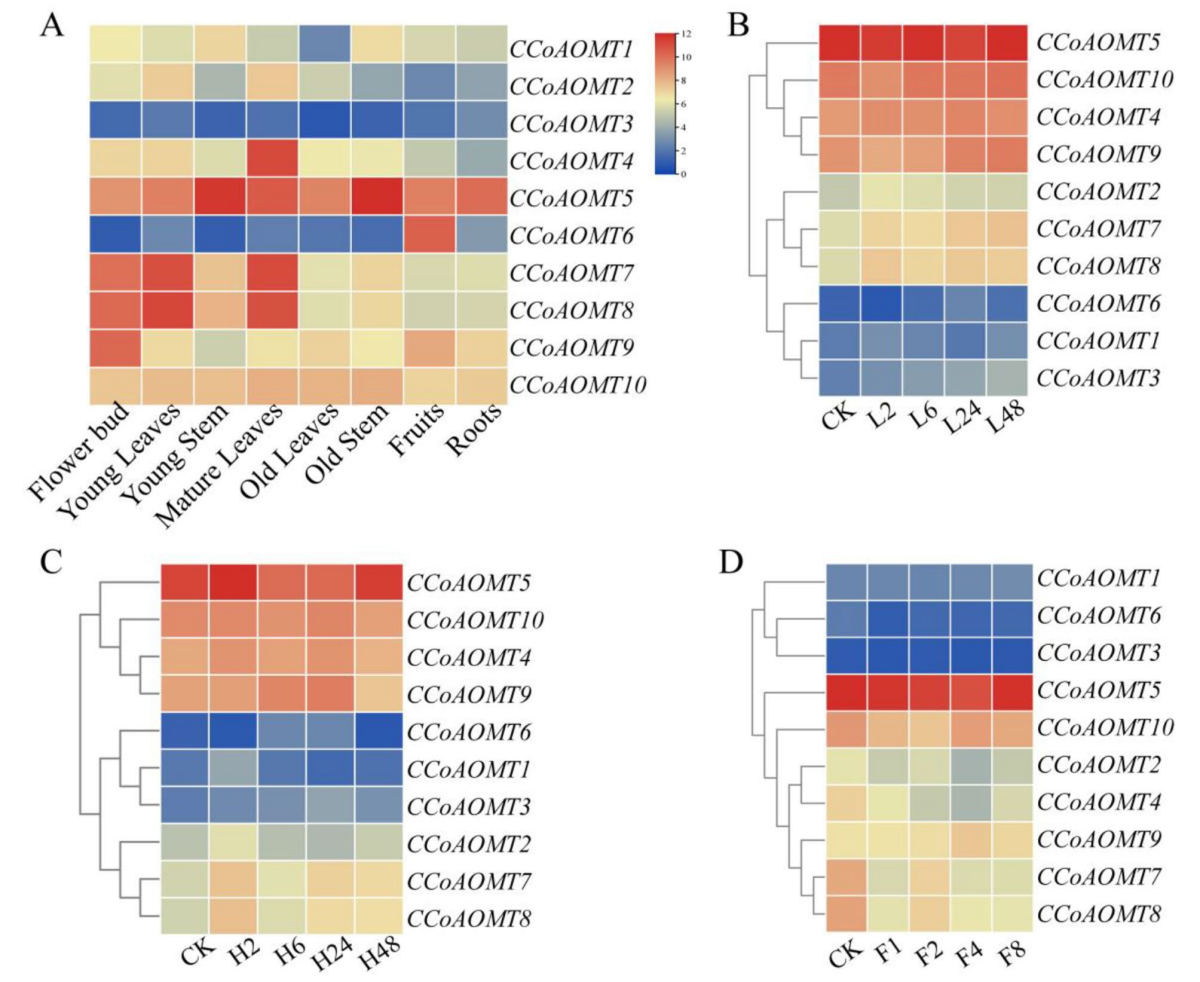
Expression patterns of *CsCCoAOMT*. (**A**) Expression patterns of *CsCCoAOMT* genes in different tissues. (**B**) Expression patterns of *CsCCoAOMT* genes in one bud and two leaves under high nitrogen treatments. (**C**) Expression patterns of *CsCCoAOMT* genes in one bud and two leaves under low nitrogen treatments. (**D**) Expression patterns of *CsCCoAOMT* genes in one bud and two leaves under fluoride treatments. The data were converted to log_2_FC (FC, fold change) and the heat map was used to represent the responsiveness of the *CsCCoAOMT* genes. Blue and red represent downregulated and upregulated genes under different tissues and treatments, respectively. Each point represented the mean values of three independent biological replicates

We examined the tissue-specific expression patterns of *CsCCoAOMT* in 8 different tissue samples of the ‘HuangDan’ cultivar. As depicted in Fig. 3A, the transcription abundance of each *CsCCoAOMT* gene varied across the different tissue samples. Notably, *CsCCoAOMT5* showed high expression levels in all tissues, particularly in the roots and stems. *CsCCoAOMT1*, *CsCCoAOMT2*, *CsCCoAOMT3*, and *CsCCoAOMT6* exhibited low expression levels in various tissue samples, while *CsCCoAOMT6* was highly expressed in fruits. Moreover, *CsCCoAOMT7* and *CsCCoAOMT8* demonstrated similar expression patterns, with higher expression levels in leaves and buds. *CsCCoAOMT4* displayed the highest expression level in mature leaves, and *CsCCoAOMT9* and *CsCCoAOMT10* were expressed in all tissue samples.

### Expression patterns of *CsCCoAOMT* under different exogenous treatments

As a plant with a preference for ammonia, the tea plant exhibits efficient absorption of ammonium nitrogen in the soil [31]. In this study, we investigated the expression pattern of *CsCCoAOMT* genes under varying nitrogen levels, including high nitrogen (HN), low nitrogen (LN), and fluoride (F) treatments. The results revealed the variation in the expression patterns of *CsCCoAOMT* members in response to different exogenous treatments. Under LN and HN treatment (Fig. 3B and C), *CsCCoAOMT4*, *CsCCoAOMT5*, *CsCCoAOMT9*, and *CsCCoAOMT10* displayed higher expression levels, while *CsCCoAOMT1*, *CsCCoAOMT3*, and *CsCCoAOMT 6* exhibited lower expression levels. *CsCCoAOMT2*, *CsCCoAOMT7*, and *CsCCoAOMT8* demonstrated specific expression patterns, with their expression levels increasing at 2 h after HN and LN treatments and then decreasing with longer treatment durations (6–48 h). However, the expression of *CsCCoAOMTs* was minimally affected by LN and HN treatment.

It is noteworthy that the fluoride content in tea leaves is several-fold higher compared to other plants [32]. Under fluoride treatment (Fig. 3D), the expression levels of *CsCCoAOMT2* and *CsCCoAOMT4* exhibited a downward trend. Similar to exogenous nitrogen treatment, *CsCCoAOMT1*, *CsCCoAOMT3*, and *CsCCoAOMT6* maintained low expression levels. Following 4 days of fluoride treatment, the expression of *CsCCoAOMT9* peaked, whereas *CsCCoAOMT7* and *CsCCoAOMT 8* showed upregulated expression levels after 2 days of treatment.

**Fig. 4:**
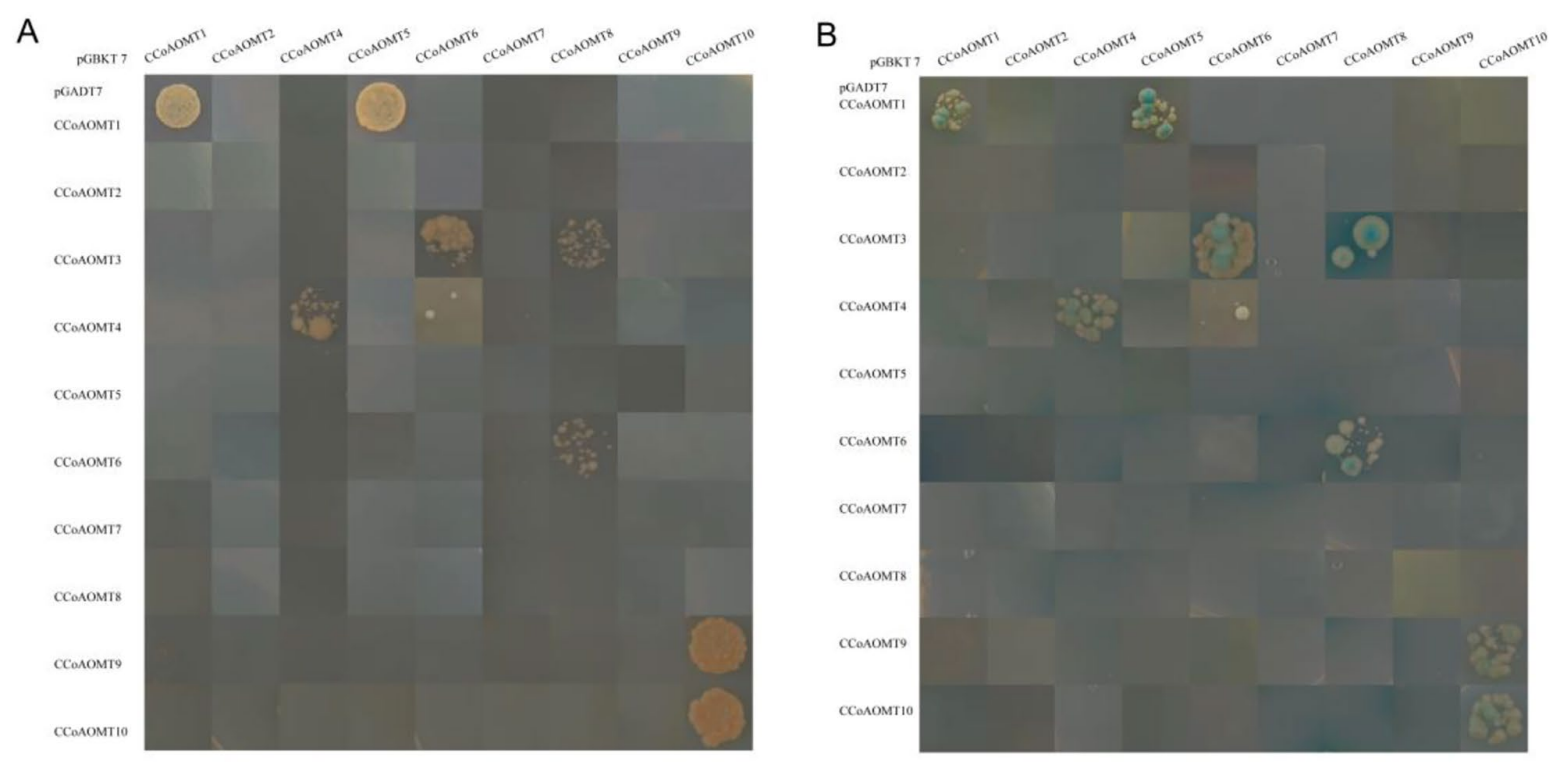
Protein-protein interactions between CsCCoAOMT proteins by yeast two-hybrid assay. (**A**) The hybrid yeast cells were grown on an SD/-Trp-Leu-His-Ade medium. (**B**) The hybrid yeast cells were grown on SD/-Trp-Leu-His-Ade + X-*α*-Gal medium

### Protein interactions of CsCCoAOMT

To reveal the protein interaction abilities of CsCCoAOMT, we conducted yeast two-hybrid analysis on 10 cloned members. The full-length coding sequences of the 10 genes were inserted into the yeast pGBKT7 vector to assess the presence of self-activation in this system. The “AH109” strain was transformed with the recombinant plasmid and empty pGADT7 vector. Our results revealed that only CsCCoAOMT3 exhibited self-activation (Fig. S1).

Yeast hybrids were then generated between these pairs of CsCCoAOMT (Fig. 4). All yeast strains grew normally on DDO (SD/-Trp-Leu) medium (Fig. S2). However, only strains containing positive dimers were able to grow on the QDO/X (SD/-Trp-Leu-His-Ade/X-*α*-Gal) medium. Three homodimers were identified in our study, including CsCCoAOMT1/1, CsCCoAOMT4/4, and CsCCoAOMT10/10. Furthermore, we identified heterodimers between CsCCoAOMT members, namely, CsCCoAOMT1/5, CsCCoAOMT3/6, CsCCoAOMT3/8, CsCCoAOMT4/6, CsCCoAOMT6/8, and CsCCoAOMT9/10. In addition, a weak interaction was observed between CsCCoAOMT4AD×CsCCoAOMT6BD. In conclusion, our Y2H analysis identified 9 pairs of interacting proteins involving CCoAOMT.

**Fig. 5:**
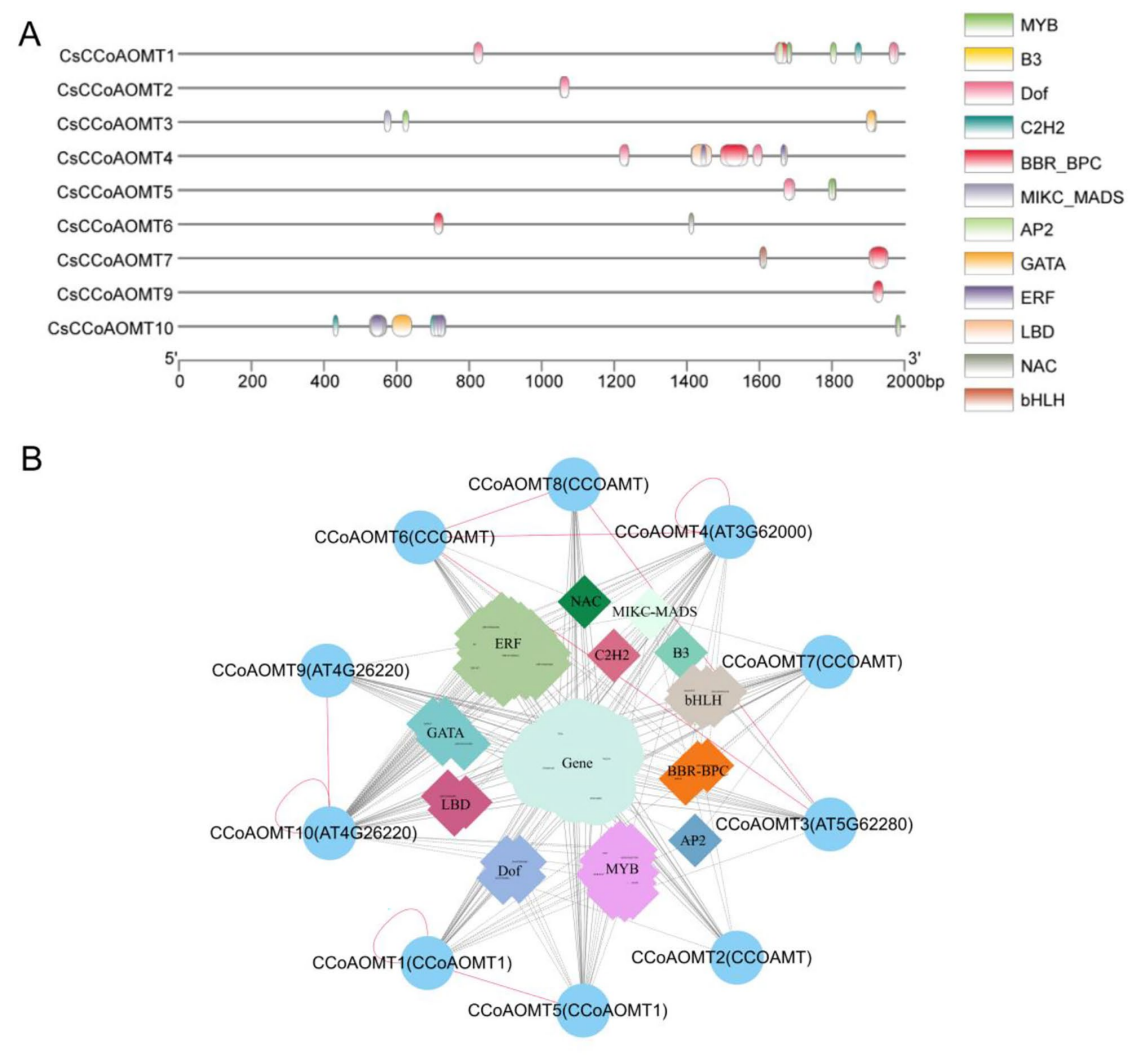
Transcription factor binding site prediction and protein interaction regulation network of HD-*CsCCoAOMT*. (**A**) Transcription factor binding sites predicted in the promoters of HD-*CsCCoAOMT*. (**B**) Protein interactions and regulatory networks of HD-CsCCoAOMT. The outermost ring represents HD-CsCCoAOMT in ‘HuangDan’. The circles represent functional genes and the diamonds represent transcription factors, different color blocks represent different families of transcription factors, the dotted line represents the interaction between genes, the solid line represents the regulation of genes by transcription factors, and the red line represents preliminary validation

### The interaction and regulation network of *CsCCoAOMT*

To investigate the regulation of *CsCCoAOMT* expression by transcription factors (TF), we utilized the PlantTFDB database to identify TF binding sites on promoters. Our analysis revealed that 12 TF families (*MYB*, *B3*, *Dof*, *C2H2*, *BBR-BPC*, *MIKC-MADS*, *AP2*, *GATA*, *ERF*, *LBD*, *NAC*, and *bHLH*) potentially bind *CsCCoAOMT* promoters, which covers 16, 6, 12, 5, 12, 9, 3, 10, 51, 6, 1 and 6 members. The highest number and variety of TFs were identified in the promoters of *CsCCoAOMT10*, while *CsCCoAOMT8* did not exhibit any TF binding sites (Fig. 5A).

We obtained a protein interaction regulatory network of CsCCoAOMT using a combination of the Y2H assay, PlantTFDB database, and STRING analysis. Specifically, we compared ten CsCCoAOMT to five AtCCoAOMT in *Arabidopsis*. A total of 27 functional genes were predicted to interact with CsCCoAOMT, as well as the presence of 77 TFs that might regulate *CsCCoAOMT* expression (Fig. 5B). Notably, these functional genes are primarily associated with lignin biosynthesis, including *4CL*, *HCT*, and *C4H*.

## Discussion

CCoAOMT is a protein that possesses a complete AdoMet MTAses domain and is capable of catalyzing the *O*-methylation modification of various compounds in plants. It plays a crucial role in the biosynthesis of lignin, flavonoids, and phenylpropionic compounds. Several *CCoAOMT* genes from different plant species, including *Arabidopsis* [20], *Marchantia paleacea* [33], and *Populus* [15], have been identified and characterized. However, little information exists regarding *CCoAOMT* in *Camellia sinensis* [34, 35], and to the best of our knowledge, the interaction between CsCCoAOMT members in *Camellia sinensis* has not been reported.

### Bioinformatics analysis of the *CsCCoAOMT* family

In this study, we employed bioinformatics methods to identify 10 *CsCCoOAMT* genes in ‘HuangDan’ tea plants and subsequently named them *CsCCoAOMT1*-*CsCCoAOMT10*. Majority of these genes exhibited isoelectric points below 7. Our bioinformatics analysis revealed that *CsCCoOAMT* genes displayed high similarities in terms of amino acid sequences, gene structures, and conserved motifs. This suggests that while some differences exist among members of the *CsCCoAOMT* gene family, they remain relatively conserved during evolutionary processes. This implies both functional similarity and differentiation among *CsCCoAOMT* genes, highlighting their synergistic roles in regulating plant growth and development.

Based on phylogenetic analysis, CCoAOMT can be divided into two subgroups. AtCCoAOMT1 was classified into the Ia branch, which has been confirmed to be involved in the lignin biosynthesis in *Arabidopsis* [36]. CsCCoAOMT1 and CsCCoAOMT5 displayed a closer evolutionary relationship with AtCCoAOMT1, indicating their potential involvement in lignin biosynthesis. AtCCoAOMT6 was classified into branch Ib and has been shown to participate in the biosynthesis of phenylpropanoid polyamine polymers in *Arabidopsis* flowers through the encoding of tapetal O-methyltransferase [37]. Furthermore, in vitro, enzymology studies have demonstrated the catalytic activity of AtCCoAOMT6 towards caffeoyl CoA, caffeoyl glucose, chlorogenic acid, and polyamine conjugates. AtCCoAOMT7 has been confirmed to play a role in phenylpropane and flavonoid biosynthesis, exhibiting a strong preference for the para methylation of flavanone and dihydroflavonol [38]. We speculate that CsCCoAOMT2, CsCCoAOMT6, CsCCoAOMT7, CsCCoAOMT8, CsCCoAOMT9, and CsCCoAOMT10, which fall within the same branch as AtCCoAOMT7, may participate in flavonoid biosynthesis. CsCCoAOMT3 exhibits a distant evolutionary relationship with other CsCCoAOMT members and forms an outgroup with AtCCoAOMT11. We speculate that this protein may have undergone mutations during evolution.

Since protein structure determines its function, variations in gene structures may lead to changes in protein binding conformation, thus significantly impacting gene function. The protein motif of CCoAOMT is relatively conserved, and members with high homology typically possess similar exon numbers and conserved protein motifs. Consistent with the phylogenetic tree results, the motif of CsCCoAOMT3 differed significantly from that of other members, featuring only two exons. Interspecific collinearity analysis revealed that CsCCoAOMT5 and CsCCoAOMT10 were homologous to AtCCoAOMT1 and AtCCoAOMT7, respectively. Based on these findings, we speculated that these genes may share similar functions.

### Response of the *CsCCoAOMT* family to nitrogen and fluorine treatment

Nitrogen has a significant impact on the levels of lignin and anthocyanin. When poplar roots were exposed to low-concentration ammonium nitrogen treatment (0.1 mmol/L), the expression of *PtCCoAOMT2* was inhibited, while *PtCCoAOMT4* expression was promoted in the lower stem. However, there was no significant difference in the expression of *PtCCoAOMT1* and *PtCCoAOMT2* in the upper stem under high-concentration ammonium nitrogen (10 mM) [15]. In nitrogen-restricted conditions, the expression of *AtCCoAOMT6* was significantly upregulated in the *Arabidopsis nla* mutant [39].

In our study, the expression level of the *CsCCoAOMT* gene in ‘HuangDan’ leaves under different nitrogen concentrations was detected, but the expression of gene family members didn’t change significantly, indicating that ammonium content was not the primary factor influencing the expression of *CsCCoAOMT*.

*Camellia sinensis* are known for their high fluoride content, particularly in mature leaves [32]. Following fluoride treatment, there was a general downward trend in the expression of *CsCCoAOMT2*, *CsCCoAOMT4*, *CsCCoAOMT7*, and *CsCCoAOMT8*, indicating that fluoride treatment inhibited the expression of these genes.

### Interaction relationship of the *CsCCoAOMT* family

Currently, there are limited reports on the interaction involving the CCoAOMT protein. For instance, AtCCoAOMT7 has been demonstrated to bind with *S*-adenosyl-L-homocysteine hydrolase (SAHH) and *S*-adenosyl-L-methionine synthases (SAMS), thereby influencing the ferulic acid content in cell walls by mediating SAH degradation [40]. However, there is no information available regarding the interacting proteins of CsCCoAOMT members in tea plants. Through yeast two-hybrid experiments, we identified three pairs of homodimers: CsCCoAOMT1/1, CsCCoAOMT4/4, and CsCCoAOMT10/10, as well as five pairs of heterodimers: CsCCoAOMT1/5, CsCCoAOMT3/6, CsCCoAOMT3/8, CsCCoAOMT4/6, and CsCCoAOMT6/8, and CsCCoAOMT9/10. Homodimers and heterodimers play vital biological roles in organisms. Homodimers enhance protein stability and activity, whereas heterodimers form new structures and functions by combining different protein units, thereby expanding biological functionality. In cassava, the self-association of MeMDH1 promotes malate biosynthesis and confers disease resistance. The Cys330 residue is directly associated with MeMDH1 self-association and enzyme activity [41]. Peach exhibits a wide range of homodimer and heterodimer patterns among TIFY members, enabling their significant involvement in various biological processes [42]. Given the broad involvement of CCoAOMT in the biosynthesis of secondary metabolites such as lignin and flavonoids in plants, and its participation in the biosynthesis of EGCG3"Me in tea plants, we speculate that the homo- and heterodimer patterns help to enhance the enzymatic catalytic activity of CsCCoAOMT, facilitating more efficient synthesis of various secondary metabolites and playing pivotal roles in different biological processes.

Based on our experimental data, we strongly believe that *CsCCoAOMT4*, *CsCCoAOMT5*, *CsCCoAOMT9*, and *CsCCoAOMT10* play important roles in tea plants. Firstly, qPCR results demonstrated a high expression trend of these four members in various tissues and under different exogenous treatments. Additionally, the Y2H results indicated the interaction of these four members with CCoAOMT. Consequently, we conclude that *CsCCoAOMT4*, *CsCCoAOMT5*, *CsCCoAOMT9*, and *CsCCoAOMT10* play significant roles in the anabolism of polyphenols in *Camellia sinensis*.

As a key factor affecting the quality and grade of tea, the tenderness of fresh leaves is inversely correlated with the lignin content. From the perspective of plant development and stress resistance physiology, lignin promotes the growth and development of tea plants and enhances their resistance [43, 44]. However, considering the quality of tea beverages, high lignin content leads to increased leaf content, which affects processing and product quality. CCoAOMT plays a crucial role in lignin biosynthesis and secondary cell wall formation in higher plants. In addition, CsCCoAOMT can methylate EGCG to produce *O*-methylated catechins [9, 45], which are reported to have stronger anti-allergic and anti-obesity effects compared to EGCG. Thus, the *CsCCoAOMT* gene family significantly contributes to the quality and grade of tea products and their associated health benefits.

## Materials and methods

### Identification of *Camellia sinensis CCoAOMT* genes

The HMM profile of the CCoAOMT conserved structural domain (PF01596) was searched and downloaded from the Pfam database (http://pfam.xfam.org/). The search command in HMMER 3.0 software and the HMM profile of the CCoAOMT conserved structural domain were used to search the ‘HuangDan’ [46]protein files (1e^− 20^) (http://tpia.teaplants.cn/). After removing redundant IDs, these candidate CCoAOMT protein sequences were verified by SMART (http://smart.embl-heidelberg.de/smart/set_mode) and NCBI CDD databases (https://www.ncbi.nlm.nih.gov/Structure/bwrpsb/bwrpsb.cgi) with default parameters. The physical and chemical properties of these protein sequences, including molecular weight (MW), theoretical isoelectric point (PI), and length of sequence, were predicted by ExPASy (https://www.expasy.org/vg/index/ Protein). These *CsCCoAOMT* genes were named according to the order of distribution on the chromosomes.

### Phylogenetic tree, conserved motif, and genetic structure analysis of the *CCoAOMT* genes

The CCoAOMT protein sequences of *A. thaliana* and other plants were obtained from NCBI (https://www.ncbi.nlm.nih.gov/) and shown in Table S2. A phylogenetic tree was constructed by the neighbour-joining (NJ) method using MEGA7.0 software (https://megasoftware.net), with 1000 bootstrap replicates [47]. The tree was visualized by the Interactive Tree of Life (https://itol.embl.de/itol.cgi). The online software MEME (http://meme-suite.org/tools/meme) was used to extract the conserved motif from the CCoAOMT protein sequences, and parameter selection was ‘selecting the number of motifs: 8’; other parameters were set to default. The integrated display of the phylogenetic tree, conserved motifs, and gene structure was carried out by TBtools(http://cj-chen.github.io/tbtools/Introduction/) [48].

### Chromosomal mapping, gene duplication, and collinearity analysis

The position information of *CsCCoAOMT* on the chromosome was obtained from GFF3 and the genome sequence file of ‘HuangDan’. The collinearity analysis within the ‘HuangDan’ genome and the synteny analysis of the ‘HuangDan’ cultivar associated with Arabidopsis and another two tea plant cultivars (‘ShuChaZao’ [49] and ‘TieGuanYin’ [50]) genomes were performed and visualized using TBtools.

### *Cis*-acting regulatory elements in the promoters of *CCoAOMT*

The promoter sequence of 2000 bp upstream of *CsCCoAOMT* genes was extracted from the *Camellia sinensis* genomic sequence and then submitted to the Plant *Cis*-Acting Regulatory Element (PlantCARE) website (http://bioinformatics.psb.ugent.be/webtools/plantcare/html) for cis-acting element analysis.

### Plant materials, nitrogen, and fluorine treatments

‘HuangDan’, as a plant material, was purchased from Anxi Qianhe Tea Garden (September 2020). Samples (Five-year-old tea seedlings) for tissue-specific expression pattern analysis: 9 tissue samples (flowers, buds, fruits, young leaves, mature leaves, old leaves, young stems, old stems, and roots) were collected from the *Camellia sinensis* cultivar ‘HuangDan’. After harvest, all samples were quickly frozen in liquid nitrogen and stored at -80 °C until use.

The tea seedlings (one-year-old tea seedlings) used for the exogenous treatment experiment were cultured in water. After a month of normal and stable growth, (NH_4_)_2_SO_4_ was used as the NH_4_^+^-N source, and our study included two treatment groups: high nitrogen (9 mmol/L) and low nitrogen (0.8 mmol/L). One bud and two leaves were collected at 0 h, 2 h, 6 h, 24 h, and 48 h after treatment. We used NaF as a fluoride source to treat tea seedlings [51], NaF (1.2 mmol/L), and one bud with two leaves was collected at 0 d, 1 d, 2 d, 4 d, and 8 d. All samples were frozen in liquid nitrogen and stored at -80 °C.

Three replicates were collected for each process. All samples used for gene expression analysis were extracted and analyzed in triplicate.

### Total RNA extraction and qRT‒PCR analysis

Total RNA was extracted by an RNAprep Pure Plant Plus Kit (Tiangen, Beijing, China) according to the manufacturer’s instructions. The first strand of cDNA was synthesized using the TransScript ® II All-in-One First-Strand cDNA Synthesis SuperMix for qPCR (Transgen, Beijing, China). All cDNA samples were added to 35 µl of nuclease-free water and stored at − 20 °C before they were utilized as templates for qRT‒PCR analysis. qRT‒PCR was performed using TransStart® PerfectStart TM Green qPCR SuperMix (Transgen, Beijing, China). Specific primers (Supplementary Table 3) for the *CCoAOMT* genes were designed by Primer Premier 5.0 software. The *CsGAPDH1*(KA295375.1)was selected as the internal reference gene for the detection of gene expression [52]. Each 10 µL reaction contained 5 µL of 2×PerfectStart Green qPCR SuperMix, 0.5 µL of forward primer, 0.5 µL of reverse primer, 1 µL of cDNA, and 3 µL of RNase-free water. The amplification procedure was as follows: 95 °C for 30 s, followed by 40 cycles of 95 °C for 5 s and 60 °C for 30 s. A melting curve was performed to verify the product specificity of the PCR at the end of each reaction. The relative expression of the genes was calculated by 2^−ΔΔCt^(ΔCt(test) = Ct (target, test)– Ct (ref, test), ΔCt(calibrator) = Ct (target, calibrator)– Ct (ref, calibrator), ΔΔCt= ΔCt(test) - ΔCt(calibrator)). The gene-specific primers are listed in Table S3. Changes in the mRNA levels of related genes were normalized to those of *CsGAPDH*.

### Gene cloning and vector construction

The mixed samples of all the tissue parts of ‘HuangDan’ were used as materials, and RNA was extracted and reverse transcribed according to the instructions. *CsCCoAOMT* gene family members were cloned using ApexHF HS DNA Polymerase FS from ACCURATE BIOTECHNOLOGY(HUNAN）CO.,LTD，ChangSha，China and specific primers. The amplified target gene was constructed on pGADT7 and pGBKT7 linearization vectors by the In-Fusion method and transformed into E. coli competent, screened positive clones and sent to Sangon Biotech for sequencing. Plasmids were extracted from the bacterial solution with correct sequencing using the Mini Plasmid Kit (Tiangen, Beijing, China) for the yeast two-hybrid experiment [42].

### Auto-activation detection of the tea tree CCoAOMT family

pGADT7-CCoAOMT + pGBKT7 and pGBKT7-CCoAOMT + pGADT7 were transformed into yeast strain AH109, spread on DDO (SD/-Trp-Leu), and cultivated at 30 °C for 3–5 days. Positive colonies were detected by PCR. After PCR identification, they were spotted on DDO (SD/-Trp-Leu), QDO (SD/-Trp-Leu-Ade-His), and QDO/X (SD/-Trp-Leu-Ade-His + X-*α*-Gal) to verify whether there was self-activation activity and whether the reporter gene could be activated.

### Yeast two-hybrid verification of the interaction of CCoAOMT family members

pGADT7-CCoAOMT and pGBKT7-CCoAOMT were transformed into yeast strain AH109 and cultured at 30 °C for 3–5 days, and positive colonies were picked for PCR detection. After PCR identification, they were spotted on QDO (SD/-Trp-Leu-Ade-His) and QDO/X (SD/-Trp-Leu-Ade-His + X-*α*-Gal) to verify the interaction.

## Conclusions

In the current study, we identified 10 *CsCCoAOMT* genes from *Camellia sinensis* and conducted a comprehensive analysis of their gene structure, domains, and conserved motifs. Our research clarified the evolutionary relationship between CsCCoAOMT and CCoAOMT from different plant species. Through promoter analysis, we discovered the potential roles of *CsCCoAOMT* genes in light signaling and hormone response. Moreover, we revealed the variation of distinct patterns of gene expression across various tissues and under exogenous nitrogen and fluoride treatments. Notably, we found previously unreported interactions among CsCCoAOMT members. Overall, this investigation provides novel insights for the future characterization of CsCCoAOMT genes.

### Electronic supplementary material

Below is the link to the electronic supplementary material.

Supplementary Material 1

Supplementary Material 2

## Data Availability

The mRNA and protein sequences reported in this paper are appearing in the TPIA(http://tpia.teaplants.cn/). under the accession numbers HD.01G0032320, HD.03G0010040, HD.03012642, HD.05G0001190, HD.06G0019340, HD.03G0000700, HD.06002937, HD.03G0010060, HD.12G0019390, and HD.12G0019400. *Cis*-elements were obtained from PlantCARE database (http://bioinformatics.psb.ugent.be/webtools/plantcare/html/). The *CsCCoAOMT* family expression data were generated by qRT-PCR and were available from the corresponding authors when needed. All other data supporting the results are included within the article and its Additional files.
