# Supplementary figures and images for: Genome-wide identification, expression profiling, and protein interaction analysis of the CCoAOMT gene family in the tea plant (Camellia sinensis)
Source: BMC Genomics. 2024 Mar 4;25:238. doi: 10.1186/s12864-024-09972-y (PMC10913456; doi:10.1186/s12864-024-09972-y)

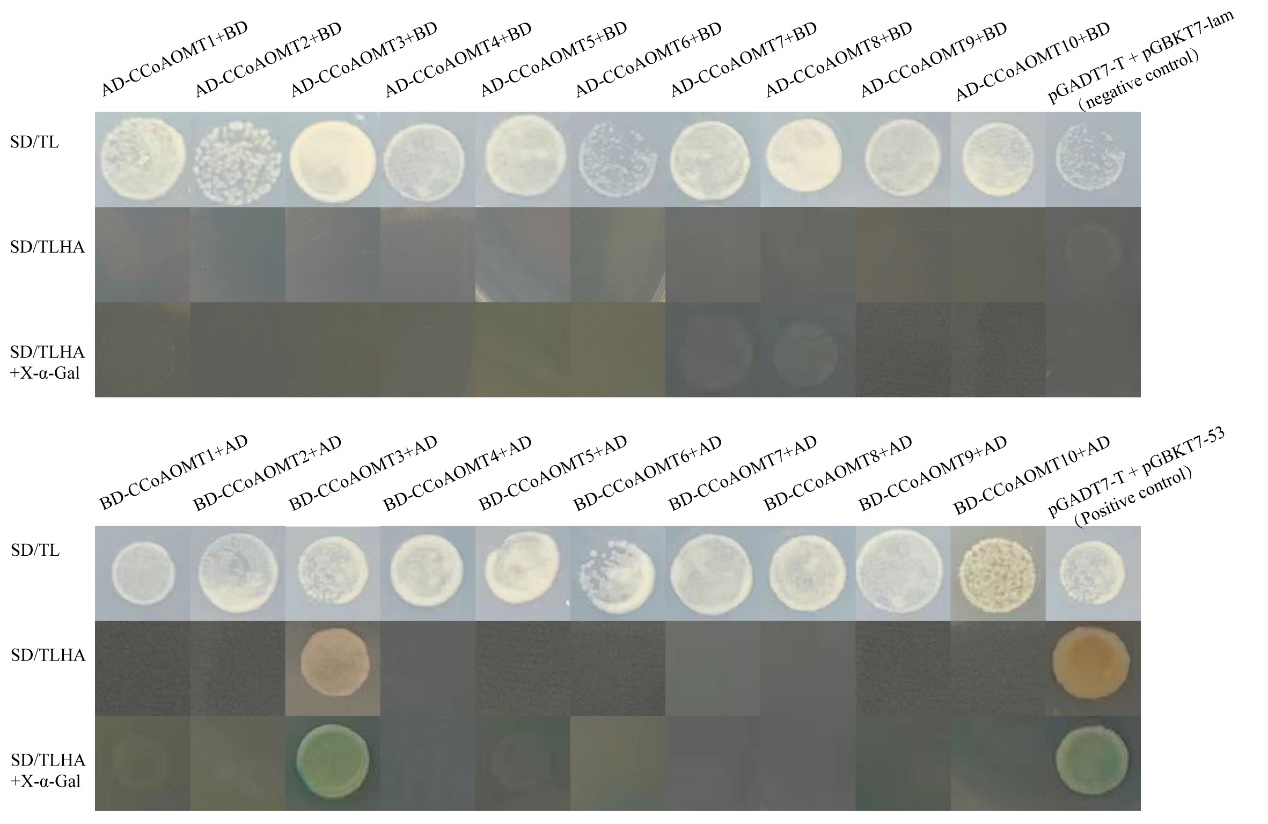


**Fig.S1.** Detection of CCoAOMT gene autoactivation


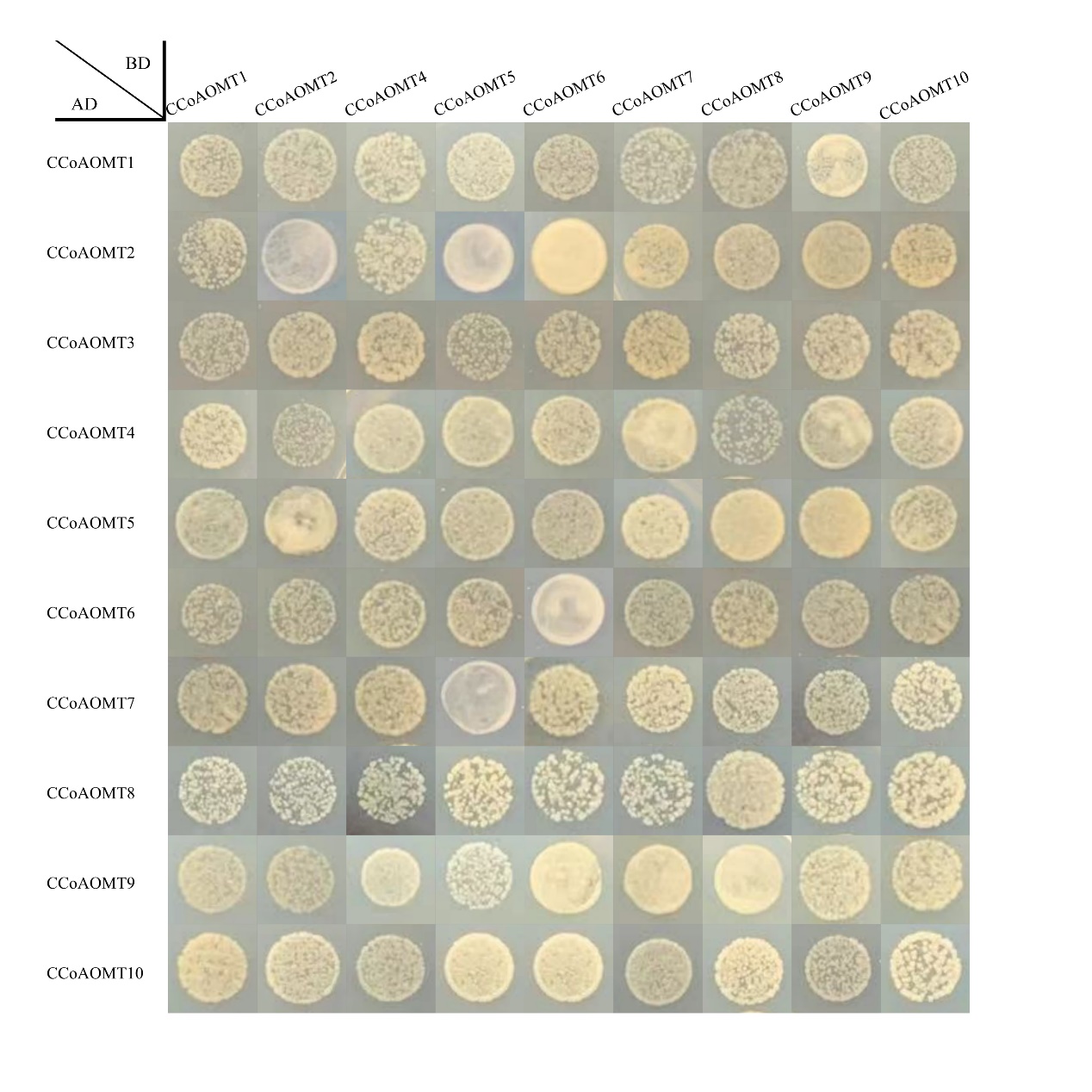


**Fig.S2.** The hybrid yeast cells were grown on DDO

Supplement: Supplementary file 2 — Supplementary Material 2 [file 12864_2024_9972_MOESM2_ESM.docx]
